# Phylogeography and Population Genetic Structure of the Ornate Dragon Lizard, *Ctenophorus ornatus*

**DOI:** 10.1371/journal.pone.0046351

**Published:** 2012-10-01

**Authors:** Esther Levy, W. Jason Kennington, Joseph L. Tomkins, Natasha R. LeBas

**Affiliations:** Centre for Evolutionary Biology, School of Animal Biology, The University of Western Australia, Perth, Western Australia; Biodiversity Insitute of Ontario - University of Guelph, Canada

## Abstract

Species inhabiting ancient, geologically stable landscapes that have been impacted by agriculture and urbanisation are expected to have complex patterns of genetic subdivision due to the influence of both historical and contemporary gene flow. Here, we investigate genetic differences among populations of the granite outcrop-dwelling lizard *Ctenophorus ornatus,* a phenotypically variable species with a wide geographical distribution across the south-west of Western Australia. Phylogenetic analysis of mitochondrial DNA sequence data revealed two distinct evolutionary lineages that have been isolated for more than four million years within the *C. ornatus* complex. This evolutionary split is associated with a change in dorsal colouration of the lizards from deep brown or black to reddish-pink. In addition, analysis of microsatellite data revealed high levels of genetic structuring within each lineage, as well as strong isolation by distance at multiple spatial scales. Among the 50 outcrop populations’ analysed, non-hierarchical Bayesian clustering analysis revealed the presence of 23 distinct genetic groups, with outcrop populations less than 4 km apart usually forming a single genetic group. When a hierarchical analysis was carried out, almost every outcrop was assigned to a different genetic group. Our results show there are multiple levels of genetic structuring in *C. ornatus*, reflecting the influence of both historical and contemporary evolutionary processes. They also highlight the need to recognise the presence of two evolutionarily distinct lineages when making conservation management decisions on this species.

## Introduction

The south-west of Western Australia is renowned for its extreme endemism, high species diversity and its threatened environments [1], [2]. This region is home to more than 5500 native plant species [1], of which 80% are endemic [3], 450 vertebrate species [1], and numerous ancient, endemic lineages of invertebrates [e.g. 4,5–7]. The diversity of taxa already recognised in the south west of Western Australia, based on prior species concepts, is nevertheless, likely to underestimate the levels of variety and evolutionarily significant diversity that is present. The issue of identifying species level subdivision is notoriously difficult [8], and with the increasing detail of molecular genetic studies, there has recently been increasing weight given to species as emergent properties of metapopulations and a more continuous notion of subdivision [9]–[11]. Alongside this is the concept of evolutionarily significant units in the context of conservation [12]. Clearly, from both a phylogenetic and a conservation perspective, it is important to identify both where subdivisions are deep enough to represent distinct species, but also where the phylogenetic and phenotypic clustering of metapopulations reveal evolutionarily significant diversity [9].

The levels of diversity, however they are delineated, have evolved in a region that is topographically subdued and in the relative absence of major vicariant forces typically used to explain speciation, such as, glaciation, tectonic or volcanic activity [13]. The diversity of flora in this region has therefore largely been attributed to the climatic fluctuations of the late Pliocene and Pleistocene [14]–[19]. By contrast, the onset of aridification of the late Miocene/early Pliocene has been suggested to have driven diversification in myobatrachid frogs [20], [21] and heath dragons [22]. The stability of this ancient landscape therefore promotes complex evolutionary histories, especially in comparison to lineages whose current distributions have arisen more recently, for example, since the Last Glacial Maximum [23].

The ornate dragon is a small (20 g) agamid lizard endemic to south-west Western Australia. It has an allopatric distribution, where the majority of the species can be found south of Jibberding through to Esperance and its offshore islands, and a smaller pocket of populations persist north of Paynes Find to Meka and Sandstone [24] (Figure 3.1). The two groups are separated by little more than 100 km. The populations in the northern pocket are phenotypically distinct from their southern counterparts. In the south, dorsal colouration of the lizards ranges from almost black to a deep brown, and males have a distinctive series of white blotches running along their back. In the northern pocket, these blotches are replaced by a solid white vertebral stripe, and the dorsal colour becomes a reddish-pink [24]. The spectral reflectance of the dorsal pattern and colouration of these lizards closely matches that of the granite, making lizards less visible to aerial predators [25]. Such phenotypic divergence is suggestive of strong selection and local adaptation, possibly enhanced by a lack of gene flow across the 100 km gap between the two groups.

The ornate dragon lizard is a habitat specialist, restricted to granite outcrops, which act as a terrestrial island system, creating discrete habitat patches that are highly variable in size and distance to the nearest outcrop [26]. We have previously shown that over a small spatial scale (five km), *C. ornatus* exhibits significant genetic structuring at distances greater than 2 km [27]. In this study, we use mitochondrial DNA (mtDNA) sequence and microsatellite data to examine the evolutionary relationships and genetic structure among *C. ornatus* populations across the species’ range. Our specific aims are 1) to determine whether the phenotypically northern populations are genetically differentiated to the southern populations and 2) examine the spatial scales over which populations become genetically distinct and whether these spatial sales vary among geographical regions.

## Methods

### Sample Collection

**Figure 1:**
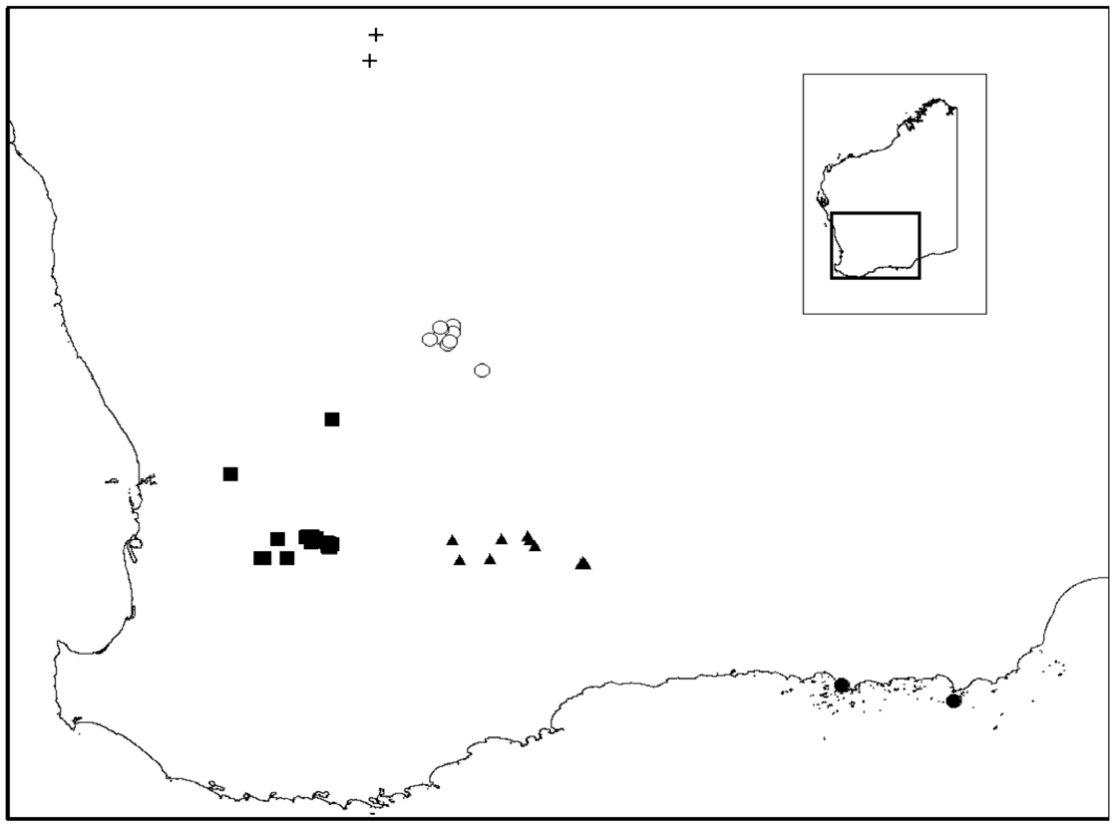
Distribution and sampling locations of *C.ornatus.* The distribution is shown by the grey shading in the inset map of Western Australia. Sampling locations for NP are indicated by crosses, NEW by open circles, SEW by triangles, S by closed circles and SW by squares.

Ornate dragons were collected from outcrops throughout the species distribution (Figure 1). Outcrops were located both in cleared agricultural land and uncleared native bushland. In total, 52 outcrops ranging in size from 0.1 ha to 175.2 ha (mean = 20.1 ha), with no less than 10 lizards (mean sample size = 23, range = 10–46), were included in this study. Outcrops were clustered into five geographic regions, Northern Pocket (NP), North-Eastern Wheatbelt (NEW), South-Eastern Wheatbelt (SEW), South-West (SW) and Southern (S) (Figure 1).

Lizards were captured by hand at first-light when they were cold and still relatively inactive by lifting exfoliated granite slabs. Each lizard was toe-clipped to provide a tissue sample for genetic analysis and then returned to its outcrop. Toe-clips were stored in 100% ethanol prior to DNA extraction. In lizards, toe and tail clipping is commonly used for identification and DNA analysis [28]–[30], and *C. ornatus* are known to lose toes as a result of predation and rock-associated injuries (LeBas pers. obs.).

### Ethics Information

This study was performed in strict accordance with the *Australian Code of Practice for the Care and Use of Animals for Scientific Purposes.* All field collection and handling practices were approved by the Animal Ethics Committee at the University of Western Australia (ref: RA/3/100/781).

**Table 1:** Levels of mtDNA variation within each of 30 outcrops.

Outcrop	Region	Haplotype(s)	*h*	π	D	*P*
N	SW	1	0.00	0.0000	0.00	1.000
S	SW	01, 02	0.40	0.0008	−0.97	0.198
Eagle	SW	01, 02, 03, 04	0.90	0.0019	−0.41	0.434
Tammar	SW	01, 02	0.67	0.0014	1.89	0.972
T	SW	1	0.00	0.0000	0.00	1.000
BBQ	SW	01, 02	0.40	0.0008	−0.97	0.201
RNR	SW	5	0.00	0.0000	0.00	1.000
Simpson	SW	5	0.00	0.0000	0.00	1.000
2Roo	SW	5	0.00	0.0000	0.00	1.000
Walton	SW	5	0.00	0.0000	0.00	1.000
SFR	SW	5	0.00	0.0000	0.00	1.000
Z	SW	5	0.00	0.0000	0.00	1.000
X	SW	06, 07	0.40	0.0004	−0.82	0.307
Y	SW	8	0.00	0.0000	0.00	1.000
Legrande	S	9	0.00	0.0000	0.00	1.000
Middle	S	10	0.00	0.0000	0.00	1.000
DD	SEW	11	0.00	0.0000	0.00	1.000
Spurr	SEW	13	0.00	0.0000	0.00	1.000
H	SEW	11	0.00	0.0000	0.00	1.000
K	SEW	12	0.00	0.0000	0.00	1.000
D	SEW	11	0.00	0.0000	0.00	1.000
G	SEW	11	0.00	0.0000	0.00	1.000
Geer C	NEW	14	0.00	0.0000	0.00	1.000
Elach	NEW	14	0.00	0.0000	0.00	1.000
Boco	NEW	14	0.00	0.0000	0.00	1.000
Falcon	NEW	14	0.00	0.0000	0.00	1.000
Moon	NEW	14	0.00	0.0000	0.00	1.000
Foggy	NEW	14, 15, 16	0.70	0.0048	1.33	0.902
Granites	NP	17	0.00	0.0000	0.00	1.000
UFO	NP	17, 18	0.67	0.0007	0.00	0.988

The haplotypes found in each outcrop are given along with the haplotype diversity (*h*), nucleotide diversity (π), and Tajima’s D with its associated *P*-value.

### DNA Extraction, Genotyping and Sequencing

DNA was extracted for microsatellite and mitochondrial analysis using the standard salting out method described in Sunnucks & Hales [31] with the exception of the incubation stage which was carried out overnight at 56°C. The microsatellite analysis was carried out according to Levy *et al*. [32]. Two loci (Co6A6 and Co9C11) are described in LeBas & Spencer [33] and the remainder in Levy *et al*. [32]. An Applied Biosystems 3730 capillary sequencer and Genemapper 3.7 software (Applied Biosystems, Foster City, California) were used to score alleles.

A subset of 30 outcrops, selected to represent each region, were used for the mitochondrial DNA analysis. For each outcrop, DNA from five adult females was sequenced. DNA from two closely related species, *Ctenophorus caudicinctus* and *Ctenophorus reticulatus* [34] were also sequenced. Tissue samples from these species (one to two individuals per species) were collected from the northern pocket of the *C. ornatus* distribution in 2000. Doublestranded PCR amplifications were executed with 25 ul of 1 × buffer (Invitrogen), 2 mM MgCl_2,_ 0.3 mM each dNTP, 0.4 uM of each primer, 1 U of Platinum Taq and approximately 20 ng of DNA. A segment of about 900 base pairs (bp) from the 12S rRNA gene was amplified with primers Co12SaF 5′GGCCCAGGACTCAAGATAACA -3′ and Co12SaR 5′TCGGCAAGTTCGTTAGGCT-3′ with the following sequencing conditions: initial denaturation at 95°C for 5 min followed by 35 cycles of 45 s at 94°C, 1 min at 56°C and 1 min at 72°C with a final elongation step at 72°C for 10 min. PCR product was run on 1.5% agarose gel for 30 min at 100 V. Samples were sequenced by AGRF (Australian Genome Research Facility) on the Applied Biosystems 3730 ×l DNA Analyzer platform.

DNA sequence data were edited using SEQUENCHER v 4.10.1 (sequence analysis software, Gene Codes Corporation, Ann Arbor, MI USA http://www.genecodes.com). Sequences were then aligned individually using MEGA v5 [35] and all alignments were checked by eye. Haplotype sequences have been lodged in GenBank (accession number: JQ627637).

**Table 2:** Characteristics of 52 outcrops and levels of microsatellite DNA variation for each outcrop.

Outcrop	Region	Outcrop size (ha)	Sample size	H_E_	AR	*F*_IS_
2Roo	SW	2.1	20	0.73	2.8	0.07
ABC	SEW	35.6	19	0.82	3.1	0.13*
BBQ	SW	4.2	20	0.79	3.0	0.06
Baladjie	NEW	32.5	31	0.74	2.8	0.15*
Bering	NEW	11.1	15	0.84	3.1	0.09
Boco	NEW	27.5	43	0.81	3.1	0.13*
D	SEW	8.0	46	0.79	3.0	0.14*
DD	SEW	5.7	25	0.83	3.2	0.09*
Dryandra	SW	2.3	20	0.72	2.7	0.06
Eagle	SW	5.1	29	0.81	3.1	0.03
EF	SEW	7.6	16	0.78	3.0	0.09
Elach	NEW	59.5	29	0.78	3.0	0.16*
Falcon	NEW	13.7	45	0.79	3.0	0.13*
FF	SW	2.9	14	0.43	1.8	−0.09
Foggy	NEW	7.3	25	0.69	2.6	0.11*
FR1	SW	9.2	14	0.79	3.0	0.06
G	SEW	56.6	23	0.82	3.1	0.11*
Geer A	NEW	1.1	10	0.80	3.0	0.11
Geer B	NEW	2.5	17	0.78	2.9	0.08
Geer C	NEW	17.1	15	0.82	3.1	0.17*
Gecko	SEW	3.2	13	0.67	2.6	0.05
Goanna	SW	0.1	20	0.84	3.2	0.06
Granites	NP	6.7	12	0.70	2.6	0.21*
H	SEW	16.1	32	0.78	3.0	0.098*
HM	SW	5.6	14	0.74	2.8	0.05
WE	SEW	43.7	23	0.78	2.9	0.13*
J	SEW	7.8	20	0.84	3.2	0.10*
K	SEW	43.7	36	0.82	3.2	0.09*
Legrande	S	1.6	27	0.46	2.0	0.04
LS	SW	3.0	22	0.77	2.9	0.06
Middle	S	175.2	10	0.43	1.9	0.25
Moon	NEW	1.3	13	0.83	3.1	0.16*
N	SW	6.3	45	0.85	3.3	0.05
P	SW	1.1	17	0.85	3.2	0.09
Q	SW	0.3	14	0.81	3.1	0.04
R	SW	1.1	15	0.83	3.1	0.04
RNR	SW	5.9	25	0.72	2.7	0.01
S	SW	0.7	23	0.82	3.1	0.02
SFR	SW	5.1	41	0.60	2.3	0.04
Simpson	SW	3.1	24	0.75	2.8	0.01
Spurr	SEW	4.4	33	0.83	3.1	0.11*
T	SW	2.1	25	0.84	3.2	0.05
Tammar	SW	3.9	24	0.81	3.1	0.03
UFO	NP	136.0	20	0.82	3.2	0.21*
V	SW	1.1	21	0.85	3.2	0.10*
W	SW	0.5	12	0.79	3.0	0.04
Walton	SW	4.7	25	0.75	2.8	0.02
WM	SW	3.3	22	0.64	2.5	0.05
X	SW	78.0	17	0.79	3.0	0.07
Y	SW	11.3	19	0.81	3.1	0.09*
Yanney	NEW	149.9	20	0.84	3.2	0.15*
Z	SW	5.3	36	0.78	3.0	0.08*

Gene diversity (H_E_), allelic richness (AR) and inbreeding coefficients (*F*_IS_) are means across all loci. Significant departures from HWE are indicated by an asterisk.

### Data Analyses

#### Mitochondrial sequence data

Departures from neutrality for each outcrop were assessed using Tajima’s [36] D statistic, as implemented in ARLEQUIN v3.1 [37]. The nucleotide diversity (π), haplotype diversity (h) and number of polymorphic sites (S) within each outcrop were also calculated in ARLEQUIN [37]. The relationship between haplotypes and their geographic distribution was explored with haplotype networks. TCS 1.21 [38] was used to create maximum parsimony networks with 95% connection limits and the geographic distribution of haplotypes was overlayed on the networks. Bayesian analyses implemented in MRBAYES 3.1.2 [39] were used to assess overall phylogenetic structure of the 12s rRNA gene in *C. ornatus*. MRBAYES calculates Bayesian posterior probabilities using a Metropolis-coupled, Markov Chain Monte Carlo sampling approach. The program jMODELTEST 0.1.1 [40] was used to determine the most appropriate model for the analyses using the Akaike Information Criterion. The GTR+G model and default priors for Markov chain Monte Carlo were adopted for the MRBAYES analysis using unique haplotypes. Four chains were run simultaneously for 400 000 generations in two independent runs, sampling trees every 100 generations. After this number of generations, the final standard deviation of split frequencies had reduced to less than 0.01 and the PSRF was 1.0 for all parameters, suggesting convergence had been reached. A burnin of 1000 trees was carried out for each independent run. A consensus tree with nodal posterior probability support was obtained and rooted with the closely related *C. caudicinctus and C. reticulatus*.

**Figure 2:**
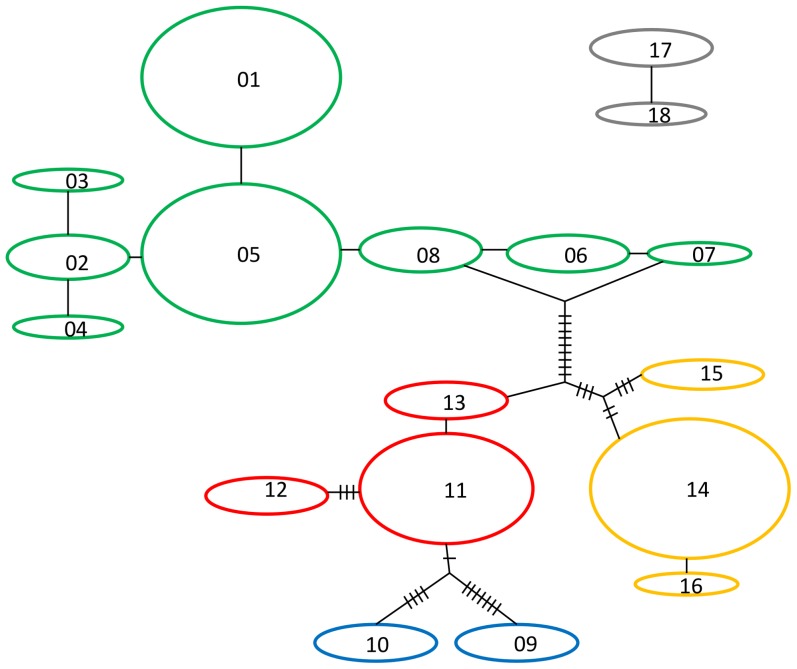
Maximum parsimony haplotype network. Each haplotype is shown as an oval, the size of which indicates the number of individuals with that haplotype (haplotypes within each outcrop are given in Table 2). Mutational steps connecting haplotypes are represented by dashes between haplotypes. NP is shown in grey, NEW in yellow, SEW in red, S in blue and SW in green.

**Figure 3:**
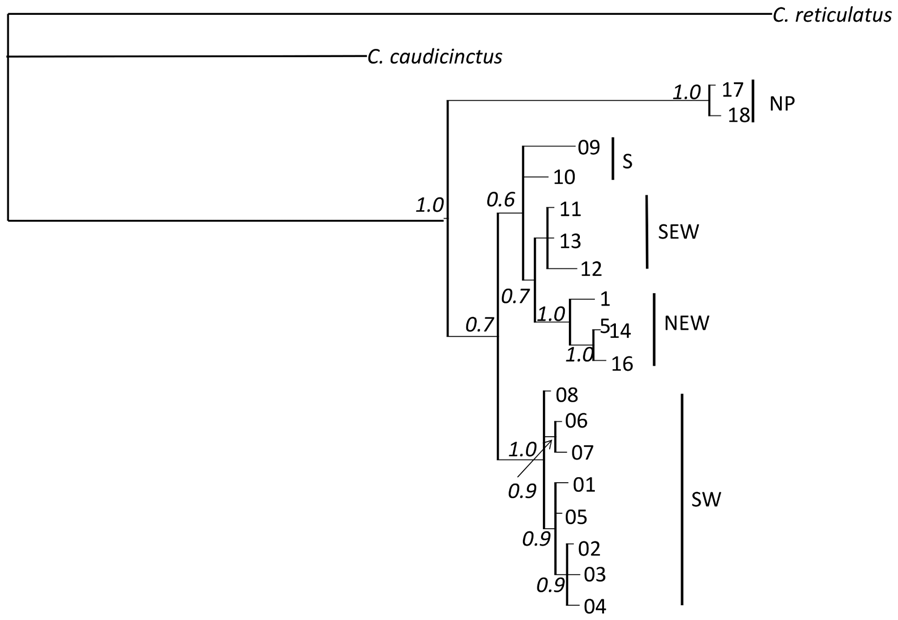
12S phylogenetic tree constructed with MRBAYES. Numbers on branches indicate posterior probabilities and regions are indicated on the right.

Pairwise genetic distances (p-distance) between and within species and each major clade were calculated in MEGA 5 [35]. Divergence times between major clades were estimated based on divergence times between *C. ornatus* and *C. caudicinctus* from Melville *et al.* [34]. Genetic structure among geographic regions was assessed using the Analysis of Molecular Variance (AMOVA) approach [41] implemented in ARLEQUIN [37]. AMOVA calculations were based haplotype frequencies and pairwise differences among haplotypes.

#### Microsatellite data

The program FreeNa [42] was used to estimate null allele frequencies for each locus, based on the expectation maximization algorithm [43]. This program creates a data set corrected for null alleles and uses it to calculate global and pairwise F_ST_ values across all loci and for each locus. As there was no difference (corrected F_ST_ = 0.15 95% CI = 0.14–0.17, uncorrected F_ST_ = 0.16 95% CI = 0.14–0.17) between these corrected F_ST_ values and the uncorrected values, the original data set was used for all remaining analyses.

The presence of linkage disequilibrium (LD) was tested for using FSTAT 2.9.3 [44]. When each outcrop was assessed individually, 14 had up to two pairs of loci in LD. Because there was no consistent pattern of LD across outcrops, all loci were retained for the remaining analyses.

**Figure 4:**
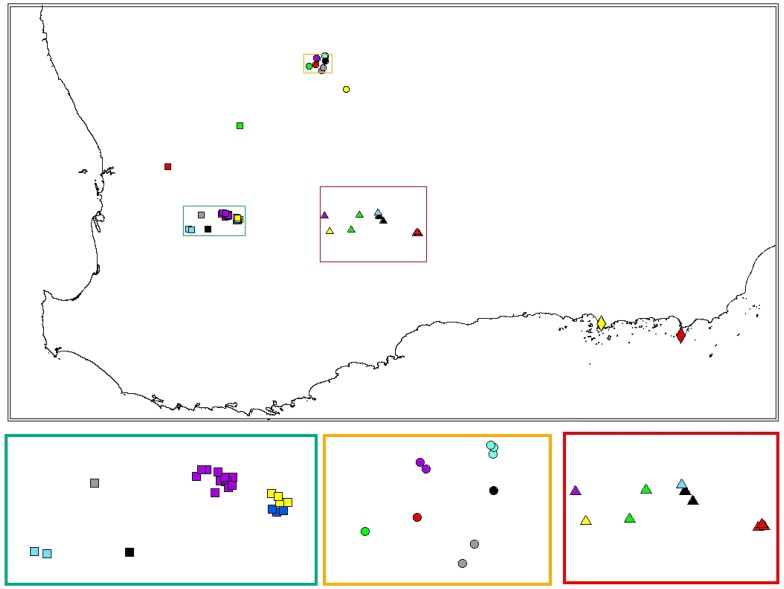
Bayesian population assignment for the southern clade generated using the software program GENELAND. Each region is indicated by a different symbol: NEW by circles, SEW by triangles, S by diamonds and SW by squares. Within each region, each cluster is represented by a different colour.

**Figure 5:**
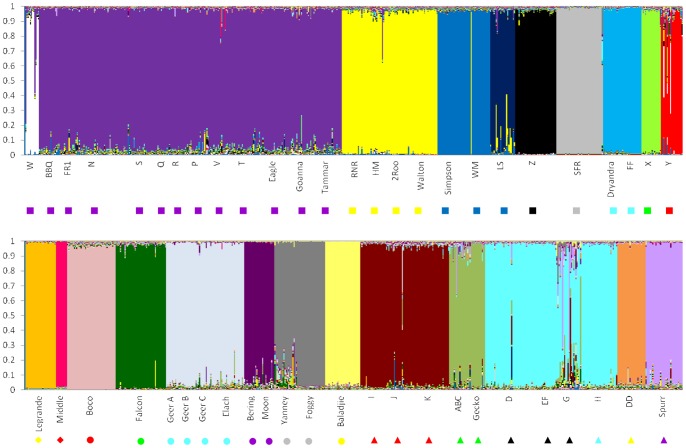
Bayesian population assignment for the southern clade generated using the software program STRUCTURE when K = 23. Each column represents a single individual and the proportion of each individual genotype that assigns to a particular cluster is shown by a different colour. Individuals are ordered according to their outcrop of origin. The symbol below each outcrop is the same as that used to represent the GENELAND cluster (Figure 4) to which the outcrop belongs. *C. ornatus* samples from the SW region are shown in the upper portion of the plot and those from the NEW, SEW and S regions, in the lower portion.

Microsatellite variation within each outcrop was measured using allele frequency data, from which the average allelic richness, inbreeding coefficient (*F*_IS_) and gene diversity (H_E_) were calculated. Genetic divergence between outcrops was estimated by calculating pairwise θ-values, an unbiased estimate of *F*_ST_ [45]. Estimates of genetic diversity within outcrops, departures from Hardy Weinberg Equilibrium (HWE) and pairwise θ-values were all calculated using FSTAT [44]. To assess the spatial genetic structure of outcrops, we analysed the correlation between genetic divergence and geographic distance to test for patterns of isolation by distance (IBD). An *F*_ST_/(1– *F*_ST_) matrix was compared with a geographical distance matrix (log km) [46], using a Mantel test (10 000 permutations) calculated with the software package VEGAN 1.17-9 [47]. IBD was tested in this way across all outcrops as well as within SEW, NEW and SW. As NP and S had only two outcrops each, mantel tests were unable to be conducted for these regions.

**Figure 6:**
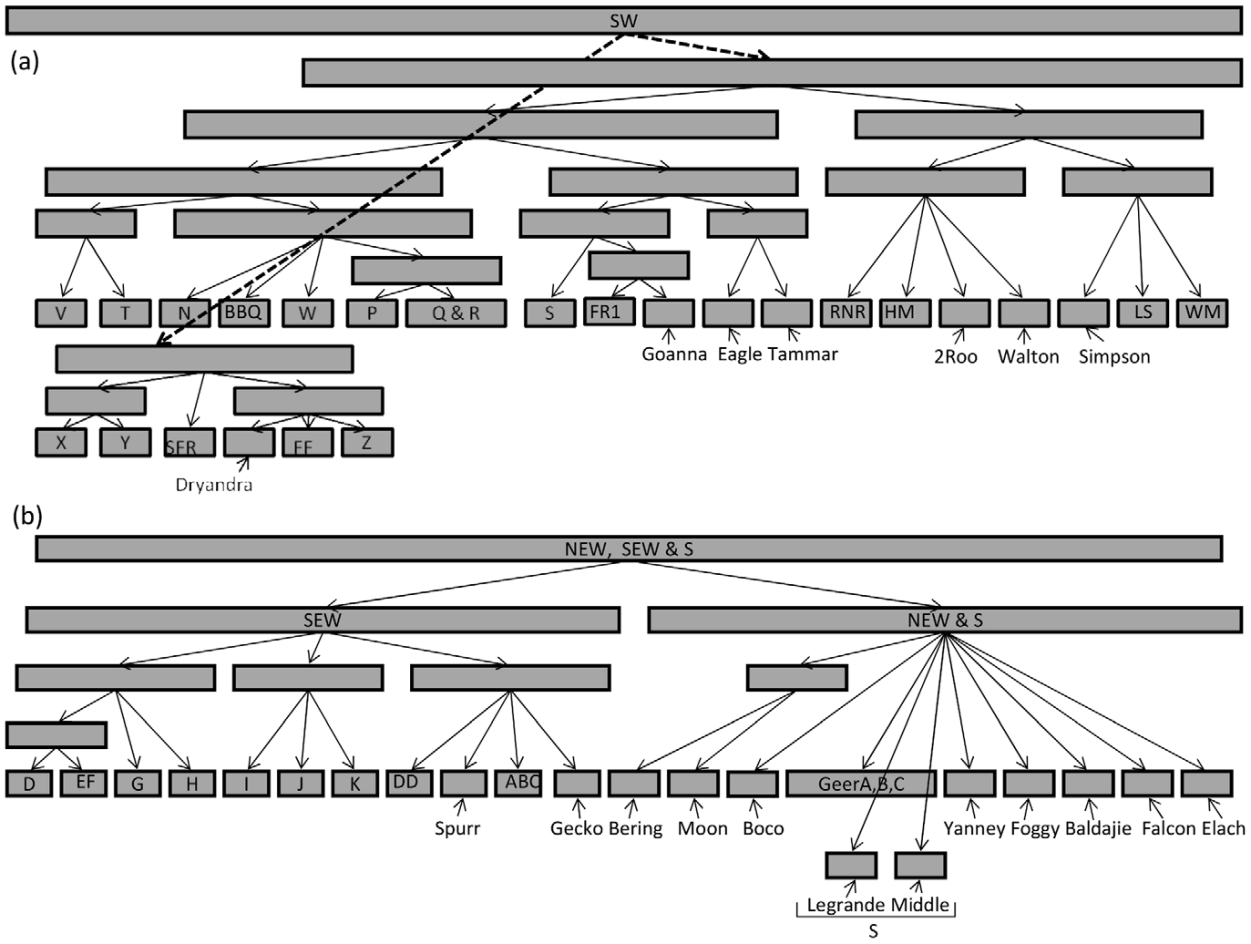
Partitioning of outcrops for the hierarchical STRUCTURE analysis. All outcrops were initially split into a western cluster (a), which consisted of outcrops from the SW region, and an eastern cluster (b) formed from NEW, SEW and S outcrops. Each outcrop is represented by a single square in the final level of the analysis. Each cluster is represented by a rectangle, the size of which corresponds to the number of outcrops within the cluster.

Population structure was also analysed with two Bayesian assignment approaches implemented using the software programs, STRUCTURE 2.3.1 [48] and GENELAND 3.1.5 [49]. Both these programs group individuals into the most likely number of clusters (K) that maximises the within cluster Hardy-Weinberg and linkage equilibria. GENELAND differs from STRUCTURE in that geographical information can be incorporated to produce more accurate inferences of population structure based on the spatial distribution of individuals.

For the GENELAND analysis, the coordinates (latitude and longitude) of each outcrop were used to run the spatial model. The uncertainty of coordinates was set at 0.4 km as this is the mean diameter of sampled outcrops. Thus, on average, any individual could have been collected within 400 m of the given coordinates. The uncorrelated and null allele models were adopted for ten independent runs of K = 1 to 52. Each run consisted of 100 000 MCMC iterations with a thinning of 100 and a burnin of 1000. The most likely number of clusters was chosen as the modal K (from each independent run) with the highest posterior probability.

Analyses involving STRUCTURE are typically assessed using the Δ*K* method as simulations have shown this method to be the most accurate estimator of the true number of populations (K) [50]. However, this method also only detects the uppermost layer of structure. Therefore the most likely K was calculated as suggested by Pritchard *et al.* [51]. We also used the hierarchical approach of Coulon *et al.* [52] to infer the number of clusters within the data set. The hierarchical analysis was run until clusters consisted of single outcrops, or K was equal to one. STRUCTURE runs were based on a model that assumed admixture of ancestry and independent allele frequencies. The assumption of independent allele frequencies in the model reduces the risk of overestimating K when allele frequencies are likely to vary significantly between samples [51]. Ten independent runs were performed for each value of K (from one to one more than the maximum number of outcrops) with a burnin of 10 000 followed by 100 000 MCMC iterations.

The significance of hierarchical partitioning of genetic structure among geographic regions was examined using the Analysis of Molecular Variance (AMOVA) approach [41] implemented in ARLEQUIN [37].

## Results

### Genetic Variation

#### Mitochondrial sequence data

Of the 145 *C. ornatus* samples sequenced for the 12s rRNA gene, 18 unique haplotypes were identified with a mean nucleotide diversity (π) of 0.00036±0.00017 and mean haplotype diversity (h) of 0.14±0.05. All of the outcrops were found to follow a neutral model of evolution (Table 1).

#### Microsatellite data

Estimates of microsatellite genetic variation were variable among outcrops (Table 2), with AR ranging from 1.8 to 3.3 (mean = 2.9) and H_E_ from 0.43 to 0.85 (mean = 0.77). The average inbreeding coefficient was 0.08 (range −0.09 to 0.25). After correction for multiple comparisons, 22 of the 52 outcrops departed from HWE with a deficit of heterozygotes. Also, two loci (CoB111 and CoD108) deviated from HWE with deficits of heterozygotes in more than ten outcrops. These deviations from HWE may be due to null alleles. The presence of null alleles for each locus within each outcrop was tested with MICROCHECKER [53]. After the removal of four loci (CoB111, CoD108, CoD3 and CoC107) that were consistently suggested to have null alleles, all outcrops were found to conform to HWE. However, a reanalysis of the data without these loci did not alter the results; thus, all loci were retained in the subsequent analyses.

### Phylogenetic Analyses

Two distinct haplotype networks were created, separating the outcrops from NP from all other regions (Figure 2). This indicates that there was less than 95% confidence in the connection between these two networks. Within the larger (southern) network, two further groups were identified, haploytpes from the SW region and those from all other regions. These two groups are separated by 13 mutational steps. Bayesian phylogenetic analyses of the 12S rRNA gene were in accordance with the haploytpe networks with NP and all other regions forming two strongly supported clades (posterior probability = 1.00, mean p-distance = 0.044) (Figure 3). Another split separating the SW region from NEW, SEW and S regions was also identified, but this was poorly supported (posterior probability = 0.68, mean p-distance = 0.016) (Figure 3).

The pairwise genetic distances for all inter species comparisons (*C. ornatus*, *C. caudicinctus* and *C. reticulatus*) ranged from 0.092 to 0.129. For all intra species and clade pairwise comparisons, p-distances ranged from 0.001 to 0.028. Pairwise p-distances between haplotypes in the northern and southern clades were intermediate to these, ranging from 0.041 to 0.047. The finding that these p-distances were distinct and did not overlap with either the intra or inter species p-distance ranges further suggests that these groups are divergent evolutionary lineages, but are not sufficiently divergent to be classified as separate species. Based on divergence times between *C. ornatus* and *C. caudicinctus* from Melville *et al.* [34], the two clades were estimated to have diverged approximately 4.26 mya.

### Population Genetic Structure

Due to the classification of NP as a clade distinct from the other regions by the phylogenetic analyses and the low number of outcrops (two) sampled from this clade, these outcrops were removed from the remaining analyses. Thus, genetic structure within the remaining 50 outcrops was analysed by STRUCTURE and GENELAND for values of K from 1–50.

Significant subdivision among outcrops was evident across all outcrops (*F*_ST_ = 0.16, 95% CI = 0.14–0.17) as well as within each region (SEW: *F*_ST_ = 0.10 (95% CI = 0.09–0.12), NEW: *F*_ST_ = 0.13 (95% CI = 0.12–0.14), SW: *F*_ST_ = 0.14 (95% CI = 0.13–0.15), S: *F*_ST_ = 0.50 (95% CI = 0.39–0.62)). Similarly, IBD was identified across all outcrops (r = 0.39, *P*<0.001) as well as within each region (NEW: r = 0.64, *P* = 0.001, SEW: r = 0.35, *P* = 0.004, SW: r = 0.51, *P* = 0.002).

The GENELAND and STRUCTURE (where K was determined by the method suggested by Pritchard *et al.* [51]) analyses revealed the most likely number of clusters to be 23. Both analyses formed similar clusters with only minor variations (Figures 4 and 5). There was no overlap between regions with all clusters consisting only of outcrops from within the same region. In general, outcrops that were closely situated to another outcrop (less than 4 km) were clustered together (Figures 4 and 5). Although two clusters in NEW contained outcrops that were more than 4 km away from the next nearest outcrop (G is 8.4 km away from D, and ABC is 23.8 km away from Gecko). Interestingly, there are other outcrops in NEW situated similar distances or even closer to neighbouring outcrops that are not assigned to the same cluster. All clusters had a small amount of admixture, mostly with neighbouring clusters. A few individuals had almost their entire genome assigned to multiple clusters, and a single individual in WM was assigned entirely to the next nearest cluster (Figure 5).

The hierarchical STRUCTURE analysis initially grouped all outcrops within the southern clade into two clusters which reflected the separation of regions by the phylogenetic analyses (cluster 1 = SW, cluster 2 = NEW, SEW and S). The SW cluster had more hierarchical structure than the eastern cluster, where outcrops formed their own clusters after only two to three levels of partitioning. Details of the levels of partitioning are given in Figure 6. Partitioning conformed to regions and at all hierarchical levels, clusters were formed from neighbouring outcrops. In general, hierarchical clustering was in line with clustering patterns from the GENELAND and STRUCTURE (where K was determined by the method suggested by Pritchard *et al.* [51]) analyses. The hierarchical analysis gave a final K of 47, where almost all outcrops formed independent clusters.

Hierarchical structuring was also found using AMOVA. A significant portion of the mtDNA and microsatellite variance was found among regions as well as among outcrops within each region, though the regional differences were much higher for mtDNA, especially when calculations were based on genetic distances among haplotypes (Table 3).

## Discussion

### Phylogenetic Analyses

The phylogenetic analysis was unable to resolve clearly whether the two allopatric groups of populations of *C. ornatus* comprise separate species. Each group was monophyletic, which is a property of the species category under the phylogenetic species concept [54]. However, the average pairwise sequence divergence between the groups (4.4%) was half of the divergence between *C. ornatus* and its most closely related species, *C. caudicinctus* (9.5%). In addition, the ranges of pairwise sequence divergences between major clades did not overlap with those within species/clades or between species. This suggests that while the northern and southern clades are independent lineages, they may not be sufficiently diverged to be considered separate species. However, the strength of this result is limited by the low number of 12S sequences available for species in the genus *Ctenophorus*. In a phylogenetic study based on all *Ctenophorus* species using sequences from three protein coding genes (ND1, ND2 and CO1), Melville *et al.* [34] found levels of sequence divergence between most species to be similar or higher than that between *C. ornatus* and *C. caudicinctus.* However, a few species exhibited divergences that were 50% lower than the sequence divergence between *C. ornatus* and *C. caudicinctus* [34]. Thus, the levels of sequence divergence found within *C. ornatus* may be sufficient to recognise the major clades as separate species. Therefore, whether there are two species currently classed as *C. ornatus* remains uncertain and more work is needed to clarify this situation. Nevertheless our study reveals that the two major clades do represent independent evolutionary lineages and, given that they also cluster by phenotype [24], care should be taken to treat them as evolutionarily distinct in future research and when making conservation management decisions.

In keeping with other phylogeographic studies on vertebrates in south-west Western Australia, the two clades were estimated to have diverged in the late Miocene to early Pliocene, approximately 4.26 mya. This time period coincides with the onset of aridity in Australia [55]. Edwards *et al*. [21] suggested that the onset of aridity during this time period isolated populations of the Myobatrachid frog, *Metacrinia nichollsi*, restricting them to refugia, and eventually causing the formation of separate lineages. This may also be the case for *C. ornatus*, as granite outcrops are thought to act as refugia during times of aridity [56]. If conditions in the environment between outcrops were too harsh, dispersal may not have occurred, causing outcrops to become isolated. However, there does not appear be a lower density of granite outcrops between the northern and southern populations.

Another possible explanation for the divergence may be the different types of vegetation in the two areas. The southern clade extends only as far as the boundary between the South-West and Eremean botanical provinces [57]. The vegetation around the granite outcrops in the northern clade is mainly *Acacia* shrubland, while in the south, eucalypt woodlands dominate [55], [57]. Even though *C. ornatus* is a granite outcrop specialist, they must travel through the intervening environment to enable gene flow between populations. It is therefore possible that different vegetation types differentially affected gene flow. Levy *et al.* [27] established that land clearing significantly reduced gene flow in *C. ornatus*, probably because it creates more open, exposed habitats with little shelter. Similarly, the sparser vegetation in the northern clade may have restricted gene flow. Pairwise *F*_ST_ for outcrops separated by similar distances (25–31 km) in the SW as those in NP (28.3 km) indicate that this may be the case. Outcrops in SW (where land clearing has been established for longer [57]) had similar or higher *F*_ST_ (mean *F*_ST_ = 0.18) than that in NP (0.17), whereas in the less cleared eastern regions, mean pairwise *F*_ST_ was lower (0.12) than in the NP. Therefore, if gene flow was affected by more sparse vegetation in the NP, dispersal between the clades could simply have become too difficult. Further, each clade could have become locally adapted to dispersing through different vegetation types, making dispersal between the northern and southern clades too difficult. A similar situation was identified in the heath dragon in south-western Australia, where allopatric populations had diverged substantially due to geographical isolation and subsequent adaptation to different habitats [22].

**Table 3:** Hierarchical analyses of molecular variance (AMOVA) apportioned among regions, outcrops and individuals.

Source of variation	% TotalVariance	*F*-statistics	*P*
Microsatellites			
Among regions	3.6	*F*_CT_ = 0.036	<0.001
Among outcrops within regions	13.2	*F*_SC_ = 0.137	<0.001
Within outcrops	83.2	*F*_ST_ = 0.168	<0.001
MtDNA (Haplotype frequencies)			
Among regions	38.3	*F*_CT_ = 0.383	<0.001
Among outcrops within regions	49.4	*F*_SC_ = 0.799	<0.001
Within outcrops	12.4	*F*_ST_ = 0.876	<0.001
MtDNA (Pairwise differences)			
Among regions	91.2	φ_CT_ = 0.912	<0.001
Among outcrops withinregions	7.2	φ_SC_ = 0.816	<0.001
Within outcrops	1.6	φ_ST_ = 0.984	<0.001

Within the southern clade, regional separation between outcrops was seen, although the regions were not divergent enough to be classified as separate clades. Further, the SW was shown to be distinct from the eastern and southern regions (NEW, SEW and S). This geographic pattern of genetic structure reflects restricted gene flow throughout the southern clade with isolation by distance (IBD) creating regional sub-clades. *Branchinella longirostris*, an anostracan endemic to freshwater shallow, ephemeral rock pools on the tops of granite outcrops in Western Australia showed a similar pattern of restricted gene flow [58]. Dispersal is likely to occur through the passive movement of eggs when rock pools are dry. Thus, even though *C. ornatus* and *B. longirostris* inhabit fragmented habitats, historical gene flow, though restricted, does take place.

### Contemporary Patterns of Genetic Variation

Patterns of IBD were identified within regions by the microsatellite analyses. IBD occurs when the divergence between populations increases linearly with increasing spatial distance between populations [59]. Patterns of IBD are commonly found in reptiles [e.g. 60,61,62] including those inhabiting landscapes modified by agricultural practices [e.g. 28,63,64,65]. This pattern suggests that in *C. ornatus* gene flow is present between outcrops, but becomes restricted as the distance between outcrops increases. In other words, outcrops within regions were not isolated from one another by complete barriers to gene flow.

The AMOVA for both mitochondrial and nuclear DNA showed there was significant genetic differentiation between regions and among outcrops within each region. These results are supported by the clustering analysis, which suggest there has been no recent gene flow between regions within the southern clade. On a finer scale, the three clustering analyses revealed largely similar patterns with neighbouring outcrops clustered together, for the GENELAND and non- hierarchical STRUCTURE approaches, or forming higher levels of structure within the hierarchical STRUCTURE analysis, and distant outcrops forming separate genetic groups. Thus, the genetic clustering patterns within *C. ornatus* support the isolation by distance analyses with distance between outcrops being important in determining levels of gene flow.

The distance at which gene flow in *C. ornatus* becomes limited, is difficult to determine as there are several conflicting pieces of evidence to consider. The GENELAND and non- hierarchical STRUCTURE analyses generally indicate that outcrops more than 4 km apart are independent genetic groups. This is largely consistent with the findings of Levy *et al.* [27] where outcrops separated by more than 2 km were classed as single genetic groups. It is interesting to note that in the few cases where outcrops more than 4 km apart were assigned to the same genetic group, they were mostly separated by native vegetation, which we have shown to be more conducive to gene flow than cleared landscapes [27]. When a hierarchical clustering analysis was performed, almost every outcrop was assigned to an independent genetic group. Such a result is not unexpected given that *C. ornatus* is restricted to granite outcrops, and therefore likely to show degree of genetic independence among populations from different outcrops.

Within outcrops, gene diversity was similar to that observed in other reptiles [e.g. 28,64–68]. Genetic variation is maintained by large population sizes and/or gene flow among populations, but these also reduce genetic divergence between populations [59]. Given that genetic divergences and genetic structure were also high among outcrops, this suggests that gene flow over short distances is sufficient to maintain gene diversity in *C. ornatus*. However, over larger distances gene flow becomes restricted causing outcrops to become divergent.

### Conclusions

The phylogeography and population genetic structure of *C. ornatus* provide an insight into the patterns and potential processes that have driven the evolution of biodiversity in the south-west of Western Australia. The timing of divergence between evolutionary lineages is in line with those in other Western Australian vertebrates [21], [22] and suggests that the onset of aridity during the late Miocene and early Pliocene may have caused the isolation of the northern portion of the species distribution from the larger southern portion. At a finer spatial scale, patterns of population genetic structure suggest that gene flow within geographical regions is restricted by distance, though there were indications that gene flow may be restricted by land-clearing as well. Thus, both historical and contemporary processes appear to influence population structure in *C. ornatus*. Finally, the presence of two distinct evolutionary lineages also highlights the need to consider intraspecific variation in future research and when making conservation management decisions on this species.
